# The Journey of the Bacterial Symbiont Through the Olive Fruit Fly: Lessons Learned and Open Questions

**DOI:** 10.3390/insects16080789

**Published:** 2025-07-31

**Authors:** Inga Siden-Kiamos, Georgia Pantidi, John Vontas

**Affiliations:** 1 Institute of Molecular Biology and Biotechnology, Foundation for Research and Technology-Hellas, 70013 Heraklion, Greece; georgia_pantidi@imbb.forth.gr; 2 Department of Biology, University of Crete, 70013 Heraklion, Greece; 3 Pesticide Science Laboratory, Department of Crop Science, Agricultural University of Athens, 11855 Athens, Greece

**Keywords:** *Bactrocera oleae*, *Candidatus* Erwinia dacicola, symbiosis, fly–bacteria interaction, dysbiosis

## Abstract

Dysbiosis is a strategy to control insect pests through disrupting symbiotic bacteria essential for their life cycle. The olive fly, *Bactrocera oleae*, has been considered a suitable system for dysbiosis, as the insect is strictly dependent on its unique symbiont *Candidatus* Erwinia dacicola. Here, we review older and recent results from studies of the interaction of the symbiont and its host fly. We then discuss possible methods for disrupting the symbiosis as a means to control the fly. Specifically, we summarize studies using microscopy methods that have investigated in great detail the organs where the bacterium resides and it is always extracellular. Furthermore, we discuss how genome sequences of both host and bacterium can provide valuable resources for understanding the interaction and transcriptomic analyses that have revealed important insights that can be exploited for dysbiosis strategies. We also assess experiments where compounds have been tested against the symbiont. The hitherto limited efficacy in decreasing bacterial abundance suggests that novel molecules and/or new ways for the delivery of agents will be important for successful dysbiosis strategies. Finally, we discuss how gene drive methods could be implemented in olive fly control, though a number of hurdles would need to be overcome.

## Simple Summary

The olive is a major crop in areas with a Mediterranean climate, constituting a major income source for farmers. The olive fruit fly, *Bactrocera oleae*, is a serious pest of the crop, and huge losses are incurred every year; it is present in most areas where olives are cultivated. The fly lays its egg in the olive, and the larvae develop there, damaging the fruit as they digest the flesh. In the unripe olive, the larvae are dependent on the symbiotic bacterium *Candidatus* Erwinia dacicola for their survival. This fact suggests that a novel strategy for olive fly control could target the bacterium; this is called dysbiosis. It would constitute an attractive alternative to the use of insecticides, as the development of resistance to the most common agents, as well as environmental concerns, requires novel methods for pest control. Here, we review the published studies of the interaction of the olive fly with its bacterium during different stages of the insect’s life cycle and of experiments to target the bacterium in the laboratory, and finally, we discuss the possibility of using genetic technologies to attack the bacterium. During the last two decades our understanding of the interaction of the fly and its symbiont has improved immensely but we are still in the beginning of exploiting this knowledge for new control strategies of the fly.

## 1. Introduction

The olive fly, *Bactrocera oleae* (Diptera: Tephritidae), is the most serious pest in olive production [1]. It is present in most areas where olives are grown, in countries around the Mediterranean Sea, as well as in the Middle East and in South and East Africa, India, and Pakistan. It has also been reported in California, Mexico, and Hawaii [2,3]. Damage by the insect incurs a huge economic cost in regions where olive cultivation is an important agricultural crop. In these areas, damage can reach 80% of the value of olive oil and 100% of some table cultivars, with 5% of total olive production in the world affected [4]; for example, the cost has been estimated at EUR 100 million in Spain annually [1]. Control of the pest in the Mediterranean basin has been largely based on the use of chemical neurotoxic insecticides, and this has resulted in the development of resistance against many of the compounds used [5]. Furthermore, the ecological effects of insecticides encourage the development of new control methods based on environmentally friendly biotechnological approaches.

**Figure 1:**
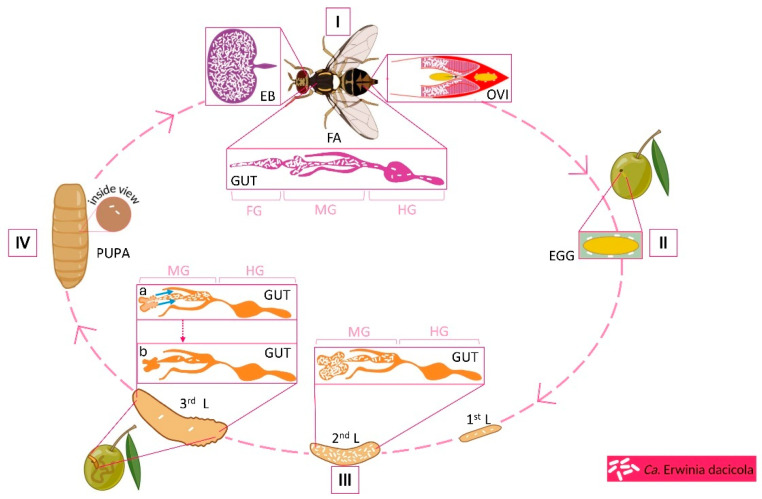
*Ca*. E. dacicola colonization and abundance during the life cycle of *Bactrocera oleae.* (I) In adult flies, the symbiont is localized in the esophageal bulb (EB), GUT, and ovipositor [OVI; present only in female adults (FA)]. Bacterial abundance is high in EB, which is entirely filled with bacteria, and in the midgut, but drastically decreases after the malpighian tubule junction, indicating lysis during transit. During oviposition, the egg is smeared with *Ca.* E. dacicola; the specific interactions enabling the adhesion to the egg surface are unknown. (ΙΙ) The female deposits the EGG into the olive mesocarp. (III) The mechanism by which the emerging first instar larva acquires the symbiont remains unresolved. During the three larval instars (L), when the larvae feed and tunnel through the olive fruit, bacterial abundance increases from the first to second instar, and they are primarily localized in the gastric caeca and midgut. As third instar larvae prepare for pupation, bacteria are discharged from the gastric caeca into the midgut, where they are lysed. (IV) In the PUPA, bacterial abundance is very low and increases substantially shortly before the emergence of the adult. The bacteria establish themselves and proliferate in the EB; the reason for this preference is not known. FG, foregut; MG, midgut; HG, hindgut. Figure created with BioRender.com accessed on 28 June 2025.

The fly is strictly dependent on the fruit of the cultivated olive tree (*Olea europaea*) and a few other closely related species for larval development. The female lays its fertilized eggs in the olive mesocarp, where the larvae develop through three instars, concomitantly destroying the quality of the fruit. After completion of the third larval instar, the larvae either leave the olive and fall onto the ground or stay in the olive, where they undergo pupation, and from the pupae, adult flies emerge, with each adult female producing hundreds of eggs during several weeks [6]. For completion of the life cycle in the unripe olives, the larvae are dependent on the presence of a symbiotic bacterium, named *Candidatus* Erwinia dacicola [7]. Figure 1 shows the life cycle of *B. oleae*, emphasizing the specific host tissues inhabited by symbiotic bacteria throughout its development. In all *B. oleae* populations, the bacterium constitutes the vast majority of the gut microbial community, although a range of other bacteria have also been reported to be present in the fly [8,9]. The fact that the fly is dependent on the symbiont has raised the prospect that one possible tool to control the olive fly population would be through targeting the bacteria. In this review, we summarize what is known about the symbiont and its interaction with the fly and its role in the life cycle of the host. We then describe possible methods for targeting the bacteria and the steps necessary to develop such intervention strategies for efficient control of *B. oleae*.

## 2. Biology of *Bactrocera oleae*–*Ca.* Erwinia dacicola Interaction

### 2.1. The Symbiont Ca. Erwinia dacicola

In 1909, Petri published a microscopic study investigating the bacteria in the fly [10]. He observed high numbers of rod-shaped bacteria in various fly organs and described their morphology but was not able to cultivate them; this has been confirmed in later studies [7,11,12]. The major species of the bacteria was identified from DNA sequencing [7], which revealed that it is a Gram-negative bacterium belonging to the family Enterobacteriaceae. It is most similar to two plant pathogens belonging to the subgroup of *Erwinia amylovora* and to another *Erwinia* sp. identified from the congeneric *Bactrocera biguttula*, which infests wild species of olives in Africa [13,14]. It was given the name *Candidatus* Erwinia dacicola and is considered an obligate symbiont, since the larvae cannot develop in unripe olives when lacking the bacterium. Since 2005, at least three haplotypes of the bacterium have been identified based on variations in their 16s rRNA sequences, and these are associated with the fly, according to its geographic distribution [3,14,15,16]. The few haplotypes of the bacterium are in contrast to the high genetic diversity of its host and are suggestive of a long-term association of host and symbiont. *B. oleae* diverged from other species about 25 million years ago and from its sister species, *B. biguttula*, about 10 million years ago. The ancestor of *Ca.* E. dacicola may have been obtained at the earlier time point, possibly acquired from a free-living bacterium residing on the plant [14]. Its low genetic diversity can be explained by strong selective pressure [3]. Genome sequences have been obtained [17,18,19]; this will be discussed further in Section 2.5.

Various other bacteria, as well as fungi, co-occur with *Ca.* E. dacicola in the gut of larvae and adult olive flies (reviewed in [20,21]). Most of these are probably acquired randomly during feeding and represent transient additions to the core microbiome associated with the fly. Several species of the genera *Pseudomonas*, *Acetobacter*, *Tatumella*, and *Enterobacter* have been highlighted for their apparent prevalence in the microbiome (see [21,22]). For the latter two, genome sequences exist that allow detailed comparisons with the obligate symbiont [17,23]. The co-occurrence of these and other bacteria with *Ca.* E. dacicola suggests that the microbiome is dynamic and may deviate spatially and temporally in species composition. The functional significance of this apparent variance is still not clear. Possibly, alterations to microbial composition in the gut align functionally with the varying diet and nutritional gaps accompanying the life cycle of the fly, eventually contributing to overall fitness. Nevertheless, despite these variations, the microbiome of olive flies remains conserved and dominated by *Ca.* E. dacicola and highly differentiated in terms of species composition when compared to that of other fruit-infesting tephritids [17,23,24].

### 2.2. Abundance of Bacteria During the Life Cycle

Fluctuations of the bacteria during the fly’s life cycle have been reported [25,26,27]. The most detailed analysis compared samples from unripe and ripe olives [26]. In larvae derived from both unripe and ripe olives, the second instar larvae had the maximum number of bacteria, which decreased in the third instar samples. The lowest number overall was from the pupal samples. In adults derived from unripe olives, the bacteria increased over time, and the females contained more bacteria compared to the males. In young adults from the ripe olive samples, the numbers increased compared to the pupae but were still very low. A recent publication reported seasonal variability of bacterial load [28]. In this study, unripe olives were collected monthly over four years, and the numbers of symbionts were determined from dissected esophageal bulbs of the adult flies using qPCR. A consistent pattern emerged, suggesting that changes in temperature and humidity correlate with bacterial load. The smallest numbers were recorded during September and December, due to low humidity and temperature, respectively.

Remarkable is the variation among individual samples in the study of Siden-Kiamos et al. [26], although each sample was pooled from five individuals. The reason for this variation is not clear, although ecological parameters could play a role. This raises cautions interpreting results from treatments intended to decrease bacterial load and necessitates careful choices of experimental conditions to minimize variation, as well as inclusion of primary data in publications.

### 2.3. The Role of Ca. E. Dacicola in the Adult and Larval Stages of the Olive Fly

The contribution of the bacterium to nutrition of adult flies has been investigated by reducing the abundance of the bacteria in the gut through continuous feeding with the antibiotic piperacillin; this did not achieve complete elimination of the bacteria but reduced the number of bacteria by more than two orders of magnitude in diets lacking protein, while less reduction was seen when feeding a complete diet [29]. In this and a further study, adult wild females treated with the antibiotic (“aposymbiotic”) were compared to untreated females; both groups were fed on different diets, and the fecundity was measured [30]. This revealed that fecundity was affected when the flies were fed on a diet missing essential amino acids or when urea was the only nitrogen source provided. Furthermore, when the flies were fed bird droppings, the aposymbiotic females’ egg production was halved compared to the flies containing the symbiont. Taken together, these data suggest that the symbiont plays a crucial role in providing the fly with essential amino acids and the utilization of nitrogen from urea to boost egg production. In the field, bacteria may allow flies to meet their need for protein by sequestering otherwise unavailable nitrogen from bird droppings (e.g., urea and other compounds) and possibly also from honeydew—an unbalanced dietary source of amino acids. Analyses of draft genomes of the symbiont has shed further light on this issue and will be discussed in detail in Section 2.6.2 [17,23]. Briefly, these results supported the notion that *Ca.* E. dacicola is able to utilize urea as a source of nitrogen but cannot recycle uric acid from waste products of the insect [23]. Furthermore, operons encoding pathways for the synthesis of some amino acids are present [17]. Taken together, these data support the notion that the symbiont is involved in the provision of nitrogen and possibly some amino acids for the needs of the adult fly, being especially necessary for the fecundity of the female fly.

The importance of the bacteria during the larval stage was first pointed out in 1966 [31,32] and further examined by the use of antibiotics [33,34,35]. Later, these findings were confirmed, and this dependency was characterized in relation to the phenology of the olive fruit [8]. Female flies derived from the wild were treated with antibiotics and allowed to lay eggs in unripe or ripe olives and compared to untreated controls. The ripe olives supported the viability of both groups of larvae, but the aposymbiotic larvae did not develop in the unripe olives. Bacterial abundance was correlated with the length of the larvae, and the authors suggested that bacteria are essential for overcoming the anti-nutritive effects of oleuropein—the olive‘s main defense compound. Oleuropein is a phenolic secoiridoid glycoside compound found in unripe olives. It is degraded in the fruit by plant enzymes, producing highly reactive toxic molecules after mechanical damage by the olive fly ovipositor during egg laying, and acts as a defense molecule against the insect [36]. According to the findings of Ben-Yosef et al. [8], due to the effects of oleuropein larvae feeding on unripe fruit, they must contend with indigestible, cross-linked protein aggregates containing little lysine, an essential amino acid for the insect. Under such restrictions, bacteria may be essential for obtaining sufficient protein. In ripe olives, where oleuropein is degraded, larvae are able to meet their protein requirements through their diet and independently of bacteria [8].

### 2.4. Association of the Symbiont with the Host

#### 2.4.1. Larval Stage

The detailed description of the symbiont–fly interaction by Petri in 1909 [10] was based on microscopy to identify the organs of the fly where the bacterium resides. His observations are very detailed and still valid. Importantly, he observed the fluctuations in the abundance of the bacteria over the life cycle and how they colonize different compartments. In the larvae, the bacteria were found in the four lobes of gastric caeca (GCs), blind sacs that are found anterior to the midgut and just below the proventriculus (PV). The bacteria are constantly dividing and are pushed out from the GCs. Just before pupation, the bacteria are expelled from the GCs through a “squeezing” of the muscles surrounding the GCs, and the bacteria are pushed into the midgut, where they are possibly rapidly lysed. A recent study revisited these observations using modern microscopy techniques [26]. This study confirmed the findings of Petri and carried out a more detailed analysis of the GCs. Importantly, it showed that the bacteria were present in the lumen of the GCs, and not residing intracellularly as suggested previously [12], and that the epithelial cells were separated from the bacteria by a caeca membrane (similar to the peritrophic membrane). Furthermore, this study revealed that the epithelial cells of the GCs with microvilli extending into the lumen had the hallmarks of metabolically active cells, which were more pronounced in the second instar samples compared to those of the third instar. The numerous bacteria in the GC lumen were observed as dividing cells but also in various stages of lysis in the second instar larvae, while the GCs of the third instar larvae were devoid of bacteria.

#### 2.4.2. Pupal and Adult Stages

Petri [10] also followed the bacteria during the pupal and adult stages. He reported that, at pupation, a few bacteria were detected in the PV and in the posterior region of the esophagus. During pupation, the midgut and the gastric caeca disappeared, and the bacteria remained in the PV and the beginning of the esophagus. In adult olive flies, the bacteria were found to associate with the host in dedicated sections of their foregut and midgut [10]. A primary organ facilitating this symbiosis was the bulb-shaped diverticulum of the esophagus, located in the head of the adult fly (i.e., the esophageal bulb, EB). The bulb was formed de novo in the emerging adult and colonized with a small number of bacteria. In the adult, the bacteria continued to multiply in the EB and were continuously expelled into the esophagus as bacterial masses discharged through a narrow opening. When passing through the midgut, the bacteria were held together in oval or polyhedral shapes, but after the joint with the malpighian tubules, very few bacteria remained, suggesting that they eventually undergo lysis as they transit to the hind section of the midgut [10]. Similar observations were reported using modern microscopy [37]. These observations suggest that the bacteria are digested in the gut and, consequently, contribute nutritionally to the fly.

### 2.5. Vertical Transmission of the Bacteria

In many insects, vertical transmission of extracellular symbionts has been described (review [38]). Vertical transmission can be achieved by several routes. Some insects deposit a jelly or capsule on the eggs, while another route is through trophallaxis, that is, exchange of bacteria via mouth-to-mouth or anus-to-mouth feeding, or the bacteria can be smeared onto the egg and then taken up by the larvae through feeding. The first option is not sustained by experimental data for *Ca.* E. dacicola. Trophallaxis has been reported in two studies on *Ca.* E. dacicola [39,40]. However, in neither of these were the offspring tested for the symbiont, and it is possible that the bacteria were only transiently associated with the flies. Another study tested the offspring in similar experiments, but this did not reveal any transmission of the bacterium from one generation to the next [41]. Thus, the stable association of insect and bacterium is dependent upon transmission via the egg at least in the laboratory setting, although it cannot be ruled out that, in nature, other routes of transmission are sometimes used.

The transmission of the bacteria from the female to the egg was analyzed in detail by Petri [10]. He described the anatomy of the ovipositor, where the vagina and rectum were joined in a common duct (cloaca). Blind diverticula (also called anal glands) lined the duct with a narrow opening toward the rectum. The symbiont was residing in the diverticula, which was further investigated by microscopy analysis [37] and later confirmed by transmission electron microscopy (EM) analysis [12]. Petri (ibid) suggested that the egg is smeared with the bacteria when passing through the common duct, and in the deposited egg, he detected the bacteria surrounding the micropylar region [10]. These observations were later confirmed in EM analyses showing bacteria inside the micropylar area [37,42] and also on the outside of the ovipositor [42]. The bacteria in the micropylar region were identified as *Ca.* E. dacicola using molecular tools [43]. Furthermore, washing the eggs with disinfectants removed the bacteria, confirming that they are present on the surface and not internalized in the egg.

### 2.6. Genomic and Transcriptomic Analysis of Host–Symbiont Interaction

#### 2.6.1. Genomic and Transcriptomic Analyses of *B. oleae*

An assembled and annotated genome of *B. oleae* with a total sequence length of 468.8 Mb is available, although many gaps still remain in the sequence [44]. The DNA sequence was complemented with RNA sequences obtained from different life stages and organs of the insect. Combined, these sequences are estimated to cover most genes (>90%). The genome, even if not complete, constitutes a valuable resource for studies of the fly and makes it possible to carry out meaningful transcriptomic and proteomic analyses.

Until now, to the best of our knowledge, no proteomics studies have been reported, but two analyses exist of differential gene expression. A comparison of wild larvae developing in unripe and ripe olives was carried out using a microarray platform [45]. This revealed that the larvae developing in unripe olives overexpressed genes involved in protein digestion as well as detoxification, reflecting the insect’s response to the harsh environment in the unripe olives. In the ripe olives, lipid metabolic processes were more pronounced, reflecting the high oil content in these olives [45]. The transcriptomic profiles of gastric caeca of second instar larvae collected from the wild compared with those of a laboratory strain shed light on processes taking place in this organ [26]. The wild sample contained the symbiont, while the latter did not, and therefore, changes in gene expression may reflect the response of the fly to the bacterium. Specifically, expression of genes encoding proteolytic enzymes and peptidases was upregulated in the field sample, which are putatively important for the digestion of proteins derived from the bacteria. Genes coding for proteins with a role in the immune response were also upregulated in the second instar wild larvae, suggesting a direct role in the interaction with the bacteria [26].

#### 2.6.2. Genomic and Transcriptomic Analyses of *Ca.* E. Dacicola

##### Genome Sequencing of *Ca.* E. Dacicola Reveals Metabolic Function Complementing Host Needs

Six assembled genomes are available in the NCBI database (https://www.ncbi.nlm.nih.gov/datasets/genome/?taxon=252393) (accessed on 19 September 2024), none of which is yet complete. The genome size is estimated to be roughly 3 Mb, and the number of genes annotated varies between approximately three to four thousand. Two analyses of the symbiont genome have been published. Estes et al. [17] combined data from multiple datasets [18,19,45] that indicated that >98.5% of the genome was covered [17]. In this study, the identification of gene products contributed to the understanding of the functions of the bacterium in relation to the host. Analysis of metabolic pathways revealed that proteins involved in the biosynthesis of a limited set of amino acids are present. Olives are rich in aspartate and arginine, and the bacterium has multiple pathways to degrade these amino acids, although there is an overall limited set of amino acid degradation pathways. The genome encodes complete sets of genes for glycolysis and the Tricarboxylic Acid (TCA) cycle but lacks the glyoxylate cycle and fermentation; a limited number of genes for enzymes for carbohydrate degradation were identified. The olive is rich in lipids, but no genes encoding proteins for lipid degradation were identified [17].

As discussed in Section 2.3, the adult flies consume diets that may contain inaccessible nitrogen (e.g., in the form of urea) but lack genes to utilize these nitrogen sources. At the same time, the gut microbiome was recorded to benefit fecundity under these conditions, probably due to *Ca.* E. dacicola [30]. The bacterium encodes proteins for nitrogen assimilation, which is tightly regulated in bacteria via nitrogen control involving several proteins, and three genes of this category were identified [17]. Furthermore, an operon, located in a putative gene island, contains genes encoding the structural proteins of the enzyme urease, accessory proteins, and the transcriptional regulator. This operon is lacking in other bacteria of the *Erwinia* genus and was possibly acquired from a free-living microorganism via horizontal gene transfer [23].

Evidence for genetic exchange of *Ca.* E. dacicola has been documented in two cases. A putative example of lateral transfer of a genetic region with genes encoding metabolic pathways was identified. A roughly 7 kb region, possibly flanked on both sides by a transposase, contained genes for metabolic pathways (genes for amino acid transport/metabolism, lipid metabolism, and a partial gene for energy metabolism). A phylogenetic analysis suggested that it may have been obtained from *Pseudomonas savastanoi* pv. *savastanoi*, a bacterium causing olive knot disease. Another example of genetic exchange is the pTA1 plasmid from the co-symbiont *Tatumella*, which is present in certain populations of *B. oleae*. It was identified in the *Ca.* E. dacicola genome from a sample, which did not contain *Tatumella*, and was putatively acquired via conjugative transfer, as the plasmid may be self-transmissible [23]. *Ca.* E. dacicola is missing several genes encoding flagellar proteins, an organelle responsible for motility [17]. The genome was analyzed for the presence of mobile genetic elements (MGEs), which were present at an unusually high fraction, a hallmark of recent establishment of symbionts [23]. The analysis further revealed that the genome size and GC/AT content of *Ca.* E. dacicola was similar to free-living relatives, in contrast to other symbionts that have a reduced genome size and a skewed nucleotide composition of their genomes. The lack of certain genes encoding proteins involved in carbohydrate and amino acid transport, metabolism, and cell motility indicates that some functions have been lost during the co-evolution of host and symbiont [17].

Taken together, the genome analysis reveals that *Ca*. E. dacicola is unable to fully complement the dietary limitations of the olive fly. However, as the genome is not yet complete, and a number of genes encoding proteins of unknown function may fulfill roles in these processes, this may be an underestimate of its metabolic capacity. Furthermore, other bacteria are also present in the fly, and these may satisfy some of the requirements of the insect during development. As an example, a comparison of *Ca*. E. dacicola to an *Enterobacter* sp., also derived from *B. oleae*, found this to be the case for certain processes important for the survival of the fly [17]. Furthermore, in some *B. oleae* populations, a co-symbiotic bacterium named *Tatumella* sp TA1 has been identified, which encodes enzymes lacking from the host and *Ca.* E. dacicola [23].

##### Transcriptomic Comparison of *Ca.* E. Dacicola Developing in Unripe and Ripe Olives

A transcriptomic comparison of the symbiont isolated from the gastric caeca of larvae developing in unripe and ripe olives was carried out before the availability of the genome sequence; for this analysis, the genome was reconstructed from pooled transcriptomic sequences [45]. The size of this reconstructed genome and the number of genes were somewhat lower compared to the genome sequence described above [17,18]. The comparison revealed overexpression in the unripe olive sample of genes involved in genetic exchange and the metabolism of aromatic, nitrogen, and nucleobase-containing compounds as well as of several genes encoding proteins with a putative role in detoxification of oleuropein; in the sample from ripe fruit, a different set of processes was noted, though with marginal statistical support.

## 3. Targeting the Symbiont for Olive Fly Control

### 3.1. Antisymbiotic Approaches–Laboratory Experiments

In *B. oleae*, only a limited number of reports have been published on experiments targeting the bacteria with antibacterial agents (Table 1). In two studies [8,29], the antibiotic piperacillin was used for laboratory experiments to explore the role of the bacteria in the adult and larval stages of the fly. In both cases, adult flies originating from the field were continuously treated for approximately three weeks, and, in the case of the larvae, they were derived from eggs laid by the treated females. This resulted in a reduction in the bacteria in the adult and larvae of roughly three to four orders of magnitude but did not achieve their complete elimination. Another study tested the antibiotic streptomycin and analyzed its effect on the total gut microbiome using DNA sequencing, but no effect on the microbiome composition was detected [9]. As *Ca.* E. dacicola cannot be cultured, it is difficult to differentiate between genetic resistance to antibiotics and the difficulty of the agent to reach the bacteria for efficient killing. Resistance to antibiotics occurs at a high frequency during treatment, and it is possible that, in the case of prolonged treatment with piperacillin, resistance occurred and thus elimination was not achieved [46]. Arguing against this hypothesis is the fact that bacterial abundance was considerably reduced, which would not be expected if genetic resistance had occurred.

Studies have also tested the effect of antimicrobial compounds on the transmission of the bacteria via the egg (Table 1) [43,47]. Both direct application to the egg and indirect application to the infested olive have been tested. The results suggest that a ten-fold reduction in bacterial load can be accomplished by the treatment of olives with dodine.

**Table 1:** Results from studies testing antimicrobial compounds on *B. oleae* to determine their effect on the symbiont *Ca.* E. dacicola.

Compounds and Treatment	Stage and Tissue Treated	Stage and Tissue Tested	Method for Determining Bacterial Abundance	Results	Comments	References
Antibiotic (100–200 µg mL^−1^ Piperacillin) Addition to food	♀ Adults	Adult: dissected esophageal bulbs	Epifluorescence microscope Statistical analysis	LD		[29]
Antibiotic (200 µg mL^−1^ Piperacillin) Addition to food	♀ Adults	3rd instar larvae: gut	HTS Bacterial counts under microscope	LD	Total bacterial community in the gut counted	[8]
Propolis 20% Copper 5% Copper Addition to food	Adults Eggs	Adult: dissected esophageal bulbs Eggs	DNA extraction PCR and DGGE analyses RT-qPCR Bioinformatics and statistical analysis	Bulbs: SD (copper compounds) SI (propolis) Eggs: NC		[48]
Propionic acid solution (0.3% PA) Mixture of sodium hypochlorite and Triton X (1:1 SHTX) Soaking	Eggs	Eggs: surface and rinse solution	DNA extraction and DGGE Stereomicroscope RT-q-PCR Scanning electron microscopy	SD (All treatments)	Bacteria are lost even when eggs are washed with water	[43]
Antibiotic (0.08% Streptomycin) Addition to food	Adults	Adult: dissected guts	DNA extraction and 16S rRNA gene amplicon library Sequencing Bioinformatics and statistical analysis	NC		[9]
Copper oxychloride (0.5%, 0.1%, and 0.02% *w*/*w*) Fungal metabolite Viridiol (0.5% and 0.1% *w*/*w*) Negative control: antibiotic (Piperacillin) Addition to food	Adults	Adult: head and abdomen	DNA extraction and qPCR Statistical analysis	Heads: All treatments (≠CO 0.02%): SD CO 0.02%: NC Abdomens: All treatments (≠CO 0.02% and Vi 0.1%): SD CO 0.02% and Vi 0.1%: NC	All treatments (≠ antibiotic and CO 0.02%): LD (total bacterial community)	[46]
Copper oxychloride (3.75%) Dodine (52.9%) Tannins (0.13%) Flavonoids (2% propolis) Micronutrient EC fertilizer [Cu (2%) + Zn (4%) + citric acid (23.8%)] Sodium hypochlorite (NaClO 1%) Soaking	Eggs Infested olives	Larvae	DNA extraction and qPCR Statistical analysis	Egg treatment: LD: NaClO SD: All other treatments Olive treatment: SD/NC: All treatments	Eggs exposed directly and indirectly	[47]
Copper nanoparticles (Cu-NPs) Copper oxide nanoparticles (CuO-NPs) Ethylenedia-minetetraacetic acid (EDTA) Copper hydroxide (Cu (OH)_2_) Deltamethrin (Decis 2.5 EC) Addition to food	Adults	Adult: thorax–head and abdomen	Scanning electron microscopy DNA extraction and qPCR Statistical analysis	SD/NC: All treatments		[49]
6-pentyl-α- pyrone 0.5%, 0.1% and 0.02% p/p (6PP) Harzianic acid 0.5%, 0.1% and 0.02% p/p (HA) Positive controls: A) Piperacillin (100 µg mL^−1^) B) Copper oxychloride (0.5%, 0.1% and 0.02% p/p) Addition to food	Adults	Adult: dissected esophageal bulbs	Cloning (*E. coli* vectors) qPCR Statistical analysis	LD: Piperacillin, SD:0.5% HA and 0.5% Cu SI: 0.1% Cu NC: 0.02% HA and 0.02% 6PP		[28]

DGGE = Denaturing Gradient Gel Electrophoresis, HTS = High Throughput Sequencing, LD = Large Decrease, >10-fold decrease, SD = Small Decrease, <10-fold decrease, NC = No Change, SI = Small Increase, ≠ different to.

Copper compounds as antibacterial agents have attracted interest, and four published studies are available; in all of these, the compounds were provided orally to adults (Table 1). Copper salts did not result in any significant reduction in the abundance of *Ca.* E. dacicola in the resulting eggs [48]. Copper oxychloride treatment resulted in a reduction in the endosymbiotic load in the adult esophageal bulb and midgut, which reached more than 50% at the higher concentrations tested compared to the control. Offspring was not tested [46]. However, in another study with the same compound, there was no significant decrease in bacterial load comparing esophageal bulbs from the treated and control samples [47]. In a fourth study, bulk and nanosized copper compounds were provided to the flies. A significant decrease in bacterial load in the treated adults was found, with a more pronounced effect on the bacteria in the esophageal bulb [49]. Taken together, the results of these studies are largely discouraging, as the effects on the symbiont of copper compounds are limited.

In conclusion, it might be possible to target the bacteria with different antibacterial agents at various stages in the life cycle. The antibiotic piperacillin has shown the most promise, but it did not eliminate the bacteria even during prolonged application. Piperacillin is only one of many antibiotics, and it is possible that agents with different modes of action could be more efficient. Field application of antibiotics that are used in humans and animals is, however, problematic as in most countries these agents are restricted for use in medical or veterinary practice. On the other hand, antibiotics may constitute important tools for studying the role of the symbiont in the laboratory, as has already been shown by the studies reported above. Copper compounds have advantages, as they are already used in agriculture, being cheap to produce and considered safe for food production, but their low efficacy is a major impediment to their use to target the symbiont. However, the fact that, so far, no agent has shown the ability to radically affect the viability of the bacterial symbiont shows that new approaches should be investigated.

### 3.2. Disruption of Symbiosis in Other Insects

Bacterial symbionts are found in many insects, where they contribute to the host’s fitness [50]. In the agricultural pest insects of the family Pentatomidae, the symbionts are considered possible targets for control; in this case, the bacteria are smeared onto the eggs, which are oviposited on crop surfaces [51]. Treatments of egg masses of *Halyomorpha halys* with antibacterial formulations have resulted in the disruption of the acquisition of the symbiont [51]. These results suggest that extracellular symbionts that are exposed to the environment could be targeted directly.

For intracellular symbionts, the situation is more complicated, as the bacteria are not exposed to the environment. In many insect pests that feed on plant sap, the bacteria reside in specialized cells called bacteriocytes. One example is Aphids that depend on the endosymbiont *Buchnera aphidicola;* in this case, the symbionts reside in a dedicated organ called the bacteriome [52]. In this system, RNAi has been suggested as a technique for controlling the host via interfering with its interaction with the symbiont. In one study, two putative immune modulators were downregulated using RNAi, which resulted in a reduction in the abundance of the symbiont [53]. Another approach used synthetic single-stranded peptide nucleic acids (PNAs) to decrease the expression of the bacterial chaperone *groEL*, which led to a significantly reduced titer of *Buchnera* [54]. These latter two studies indicate that it may be possible to target the symbiont with specific effectors that could be genetically encoded in the host insect or in the plants the insect feeds on.

### 3.3. Biotechnology Approaches for Targeting the Symbiont

Strategies to genetically modify insects to reduce their ability to transmit microbes are a subject that is intensely worked on, especially as a tool for eliminating or eradicating malaria (for a recent review, see [55]). This entails the release of gene-modified organisms that express factors that interfere with the viability of the microbe. Furthermore, the spread of the genes encoding these factors into the existing population is achieved by the use of genetic mechanisms such as the CRISPR/Cas9 system; this is called gene drive. While the subject is complex and beyond the scope of this review, it is of interest to mention the major components that would be required for such a strategy to target the symbiont in the olive fruit fly and achieve a population decline of the insect. The effector should target *Ca.* E. dacicola with high specificity and efficacy. In mosquitoes, antibacterial peptides and single-chain antibodies have been used to target the malaria parasite [56,57]. These effectors should be expressed under the control of a stage-specific promoter active in the organs where the microbe resides for maximal effect [56,57]. The gene drive system to be used should aim to spread the modified locus in the population, and the CRISPR/Cas9 system is ideal for this purpose. This short description makes it clear that the development of such a system requires detailed research of both the bacterium and the host. Furthermore, the population to be released carrying the gene drive needs to be compatible with the wild population and be competitive for mating. Other aspects that need to be taken into consideration for such a strategy are the spread of the gene drive in the population and the possibility of mutations disrupting the intended spread. Finally, the release of gene-modified insects has environmental and social implications that need to be carefully weighed against the possible gain. We consider that, for the olive fruit fly, such a strategy should be looked into, and certain aspects could be part of the research agenda already (effectors, promoters, modeling of gene drive). However, the limited resources in this field, compared to those of the mosquito, impede the rational design and implementation of gene drive, but the experience from other systems may, in the future, make this an attractive option for olive fruit fly control.

## 4. Discussion

Importantly, Tephritidae have been suggested as ideal candidates for targeting symbiosis as a means to control the insects [51]. However, the difficulties in the development of such strategies necessitate deeper knowledge of the interaction of the host with the symbiont. In the following, we highlight some research questions that we consider to be critical for understanding symbiosis in the olive fruit fly.

1. What is the critical function of the symbiont in the larval stage? The essential role of *Ca.* E. dacicola in the larvae developing in unripe olives is not yet clarified. One possibility is the synthesis of amino acids by the bacteria, which are made available to the host by the digestion of the bacteria, thus overcoming the nutritional restriction by oleuropein. This notion is supported by reports that lysis of the bacteria takes place in the gastric caeca and that genes encoding digestive proteases are differentially expressed in larvae harboring the bacterium compared to aposymbiotic samples [26]. Alternatively, the bacterium may directly contribute to the detoxification of oleuropein in larvae, thus increasing the availability of the fruit’s protein. Other possibilities include providing precursors for molecules secreted by the larvae, which ultimately deactivate oleuropein. Symbiotic bacteria of many insects play a direct role in the defense against plant toxins, utilizing a multitude of pathways (for a review, see [58]), and this aspect could be investigated for *Ca.* E. dacicola using, as a starting point, the genome sequences. Metabolites in the caeca could also be directly identified by metabolomic analyses.
2. What is the role of the symbiont in the adult? The current evidence points to the function of the symbiont in supplementing the poor nutrition of the fly, especially the lack of a nitrogen source [29,30]. This is supported by the fact that the bacteria eventually undergo lysis as they transit to the hind section of the midgut [10]. Other Tephritids share similar gut morphologies with *B. oleae* and also cultivate a large and metabolically active mass of bacteria, which are digested [59]. Adult olive flies and other Tephritids may be regarded as specialized to gain nutrition by digesting bacteria propagating internally; thus, they do not rely on external microbes for gaining nutrition. Similar mechanisms can be seen in the bacteria-directed digestive physiology of dipterans, such as *Drosophila* and other flies, which are evolutionary specialized for obtaining nutrition by digesting microbes; although, in this case, the microbes are obtained from the environment [60,61].
  The specific function of the esophageal bulb, which is also found in other Tephritids, is unknown. At least in *Ceratititis capitata* and *Rhagoletis pomonella*, this organ harbors bacteria [62,63]. In the olive fly, it maintains the association with the symbiont by enclosing the symbiont, and in addition, it may keep harmful microbes outnumbered. Crucial knowledge that is lacking is the specific characteristics of the esophageal bulb that allow bacterial reproduction. Proteomic or transcriptomic analyses of this organ may suggest specific proteins that are crucial for the symbiont and that may constitute targets for interventions.
3. What is the mechanism for the vertical transmission of the symbiont? In the laboratory, transmission to the next generation is strictly dependent upon maternal transfer of the symbiont [41], although trophallaxis may take place [39,40]. This should be investigated under simulated field conditions to establish whether the symbiont is stably transmitted for several generations if acquired by trophallaxis. The detailed mechanism for vertical transmission of the symbiont, for example, whether special interactions are necessary for the bacterium to adhere to the egg during oviposition, remains to be clarified. Vertical transmission has been suggested as a target for intervention [51], although complicating this issue is the fact that the process largely takes place inside the ovipositor as it penetrates the olive mesocarp. However, promising results have been reported from the treatment of the olive fruit, and new formulations may lead to improvement of penetration of the fruit [47].
  The exact mechanism of the uptake of the bacteria in the young larva is not completely resolved, as well as how they reach the gastric caeca. Imaging carefully staged eggs could provide more information. Fluorescently labeled bacteria would be ideal for identifying their position and proliferation, and this method was successfully used for the establishment of a synthetic insect–bacteria mutualism between the grain weevil and a free-living relative of the symbiont *Sodalis pierantonius* [64]. However, this would require the generation of transgenic bacteria, which would be challenging, given that the symbiont cannot be cultivated. Alternatively, an antibody recognizing the bacterial surface could be useful for this purpose.
4. How can *Ca.* E. dacicola be cultivated? It will be clear from this review that one important hindrance in studying symbiosis in *B. oleae* is the fact that the symbiont cannot be cultivated using standard media, as well as more specialized media for bacterial culture [7,12]. To be able to test growth in a systematic fashion, analysis of the genome may provide clues for critical requirements of the bacterium. Comparisons with genomes of relatives, for which complete genomes exist, within the genus could be one starting point. One drawback is that the genome is not yet complete for *Ca.* E. dacicola, and the lack of specific genes may be because the specific genome sequence is missing. Thus, completion of the sequence would be worthwhile. Alternatively, if a limited list of candidate genes is put together, based on the present genome sequences, the presence of the specific genes could be determined directly via PCR.
5. Is dysbiosis-based control of *B. oleae* possible in the field? *Ca.* E. dacicola is present through the life cycle of the host, and possibly any of the stages could be targeted to eliminate or reduce the symbiont. The egg is deposited inside the olive mesocarp, where larval development takes place. As mentioned above, the application of antibacterial compounds on the olive has shown a minor reduction in bacterial abundance. Thus, such a strategy is theoretically feasible, although it is currently not a realistic option due to the difficulty in implementing this in the field. The pupae reside in the Earth, and the olive groves could possibly be sprayed with agents that have the ability to penetrate the pupal case. A possible approach could be a combination of entomopathogenic fungi targeting the host with copper compounds to kill the bacteria. This would have the advantage that the bacterial number in this stage is very low, and it may be easier to achieve complete elimination, but the method would need careful consideration for its practical implementation. The adult stage is perhaps the easiest to target with baits containing sugar to which antimicrobials could be added. After uptake by the fly, the agents should reach the EB and gut, where the bacteria reside. This is a theoretically simple method, but there are several unknown factors that may confound such a solution. First, one would need to verify that indeed antimicrobials will reach the organs of interest and that their activity is not diminished by the environment in the digestive system. It would also be preferable for the agents to remain in the esophagus, bulb, and midgut and not be transferred throughout the fly. Detoxification mechanisms should also be taken into consideration. For all approaches, the choice of agent would also be critical, and at present, it is limited to conventional antibiotics used in humans and animals. While, as discussed above, copper has been suggested, it has not yet been shown to be able to eliminate the bacterial symbiont. However, it is possible that improved formulations could circumvent this obstacle, and a combination with other agents could improve efficacy. Although challenging, the development of new and safe agents targeting *Ca.* E. dacicola should be a priority for research on dysbiosis.

## 5. Conclusions

Dysbiosis is a fairly recent concept for insect control and yet to be established in practice (for a review, see [65]). Thus, there is little experience that could be of use for the development of such methods in olive fly control. Initially, the knowledge of the fly–symbiont interaction raised the prospect of dysbiosis as an attractive strategy for the control of *B. oleae*. The initial hope that it would be straightforward to eliminate *Ca.* E. dacicola [29] has been moderated by the published reports of such attempts (Section 3.1., Table 1). It is now clear that many issues remain to be resolved before these ideas will reach maturity. However, there are reasons to be optimistic that this approach could be realized in the future, but we would like to stress that this will be dependent on research focusing on the basic biology of the fly–bacterium interaction as well as innovative methods for antibacterial control in insects. The rapid developments in the fields of high throughput analysis (genomics, transcriptomics, and metabolomics), bioimaging, artificial intelligence, and material sciences are factors that should positively contribute to the critical knowledge necessary for the ambitious endeavor to control *B. oleae* through its symbiont.

## Data Availability

No new data were created.
